# Relative contribution of environmental and nutritional variables to net primary production of *Cynodon dactylon (Linn.) Pers* in the riparian zone of a Three Gorges tributary

**DOI:** 10.1002/ece3.6409

**Published:** 2020-05-25

**Authors:** Junjie Lin, Shuang Zhou, Dan Liu, Shuai Zhang, Zhiguo Yu, Xiaoxia Yang

**Affiliations:** ^1^ Key Laboratory of Water Environment Evolution and Pollution Control in Three Gorges Reservoir Chongqing Three Gorges University Wanzhou China; ^2^ Department of Agricultural and Forestry Science and Technology Chongqing Three Gorges Vocation College Chongqing China; ^3^ School of Hydrology and Water Resources Nanjing University of Information Science and Technology Nanjing China; ^4^ Institute of Quality Standard and Testing Technology Chongqing Academy of Agricultural Sciences Chongqing China

**Keywords:** hydrological regime, net primary production, nutrient use efficiency, structure equation modeling, Three Gorges Reservoir, Yangtze river

## Abstract

Our knowledge of fundamental drivers of terrestrial net primary production (NPP) is crucial for improving the predictability of ecosystem stability under global climate change. However, the patterns and determinants of NPP are not fully understood, especially in the riparian zone ecosystem disturbed by periodic drought–rewetting (DRW) cycles. The environmental (flooding time, pH, moisture, and clay content) and nutritional properties (soil organic carbon, total nitrogen, total phosphorus, ammonium (NH_4_^+^‐N), nitrate (NO_3_^‐^‐N), and C:N:P stoichiometry) were investigated in the riparian zone of Pengxi River‐a typical tributary of Three Gorges Reservoir (TGR). Structure equation modeling was performed to evaluate the relative importance of environmental and nutritional properties on NPP of *Cynodon dactylon (Linn.) Pers (C. dactylon)‐*a dominating plant in the riparian zone of TGR. Our results indicated that NPP was much lower under much severe flooding stress. All of these variables could predict 46% of the NPP variance. Nutrient use efficiency (NUE) was one of the most critical predictor shaping the change of NPP. Specifically, flooding stress as a major driver had a direct positive effect on soil moisture and soil clay content. The soil clay content positively affects the soil C: N ratio, which further had an indirect negative impact on NPP by mediating NUE. Overall, our study provided a comprehensive analysis of the effects of the combined effect of environmental and nutrient factors on NPP and showed that continuous DRW cycles induced by hydrological regime stimulate the decrease of NPP of *C. dactylon* by changing NUE strategies. Further research is needed to explore the responses of NPP and NUE under different land use to DRW cycles and to investigate the DRW effects on the combined effect of environmental and nutrient factors by in situ experiments and long‐term studies.

## Graphical abstract

All of these variables could predict 46% of the NPP variance; NUE was one of the most critical predictor shaping the change of NPP; DRW cycles induced by hydrological regime stimulate the decrease of NPP of *C. dactylon* by changing NUE strategies.

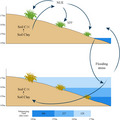

## 1 INTRODUCTION

Net primary productivity (NPP) refers to the amount of organic matter accumulated by green plants per unit area and per unit time (Zhang, Lal, Zhao, Jiang, & Chen, 2017). It is shown as the part of organic carbon fixed by photosynthesis minus the respiration consumption of plants (Ruimy, Dedieu, & Saugier, 1996). This part is used for the growth and reproduction of vegetation. As an important part of the terrestrial carbon cycle, NPP not only directly reflects the production capacity of vegetation communities under natural environmental conditions, but also is a major factor regulating the ecological process, playing an essential role in global change (Bai & Weijie, 2018). NPP has been widely used in the land‐use evaluation, regional ecological planning, vegetation growth monitoring, crop yield estimation, soil and water erosion assessment, ecological benefit assessment, etc (Piao et al., 2005).

The considerable efforts had been made to explore the spatial patterns and driving factors of NPP in variable environments. Previous studies indicated that NPP was closely associated with environmental and nutritional properties (Gholz, 1982). The exposure to drought stress decreased NPP of the invasive agronomic weed‐*Lactuca serriola* (Chadha, Florentine, Chauhan, Long, & Jayasundera, 2019). Flooding influenced physical adaptation of herbaceous plants by adjusting carbon (C), nitrogen (N), and phosphorus (P) concentration, C:N, and C:P ratios in the riparian zone of the Lijiang River (Huang, Wang, & Ren, 2019). Nutrient (N, P) use efficiency (NUE) was a ratio of photosynthetic C gains per unit of N or P invested (Castellanos et al., 2018). The interacting effect of N and P can regulate NPP, leaf stoichiometry, and NUE of *Arabidopsis thaliana* (Yan et al., 2015). The *Phragmites australis* dominated by the strategies of slow NPP and higher NUE in the tidal wetlands of the Minjiang River (Wang et al., 2015). These pioneering studies have provided convincing evidence for the mechanisms regulating NPP of perennial herbs. However, these factors may have interrelationships with each other, and little is known about the relative contribution of environmental and nutritional properties to NPP. Without this knowledge, our understanding of the driver factors of NPP and the NUE strategies to drought–rewetting (DRW) cycles remains incomplete.

Three Gorges Reservoir (TGR) is one of the largest water resource projects in the world (Wu et al., 2004). The antiseasonal DRW cycles regulated by the hydrological operation of water level interannually fluctuation from 145 to 175 m in the TGR, forming a unique artificial riparian zone with an area of approximately 349 km^2^ (Yang, Liu, Wang, Liao, & Wang, 2012). It has experienced half a year submerged on October–April in autumn–winter and another half exposed on May–September in spring–summer (Ye, Li, Zhang, & Zhang, 2011). Most of the vegetation species that settled on the riparian zone need to finish their life cycles under the limited growth period. Thus, to acknowledge the relationship of riparian zone species growth with the particular environmental condition is critical to ecological protection in the TGR.

*Cynodon dactylon (Linn.) Pers. (C. dactylon)* is a perennial herb, who is one of *the* dominant species in the riparian zone. Over 90% of *C. dactylon* can endure the oxygen deficiency and low temperature caused by winter flooding with particular root systems, and sustain to grow after long‐term flooding, and recovers rapidly in spring (Chen et al., 2019). The growth durations are different due to the distinct DRW cycles among the riparian zone altitudes of TGR (Wang, Yuan, Willison, Zhang, & Liu, 2014). Plant diversity and species richness were negatively affected by frequent flooding (Ye et al., 2020). Thus, adaptable vegetations have to pass through germination, growth, and reproduction during the dry period. The vegetation may take different life‐history strategies to adapt to the changing environment, nutrient supply, and niche differentiation (Guo, Yang, Shen, Xiao, & Cheng, 2018). The functional equilibrium hypothesis suggests that plants might maximize NPP to adapt to environmental changes by improving NUE (Marcelis, Heuvelink, & Goudriaan, 1998; Sun & Wang, 2016). Thus, the NUE strategy was crucial for *C. dactylon* to pass through the lifecycle and sustaining the stability of the riparian zone ecosystem.

In this study, the patterns and potential drivers of NPP of *C. dactylon* were explored under continuous DRW cycles by analyzing soil and corresponding vegetation samples obtained from 36 plots in the riparian zone of a TGR tributary. We also investigated the nutritional properties and synthesized hydrological data and then examined the relative importance of the physical environment (i.e., soil particle size composition, soil moisture, and flooding time), nutrition supply, and C:N:P stoichiometry properties in regulating the distribution of NPP. Specifically, we aimed to answer the following two questions: (a) How the combined effect of environmental and nutritional variables determines spatial variation of NPP and NUE in the riparian zone? (b) Which is the dominant factor that driving differences in NPP and NUE under continuous DRW cycles? We hypothesize that (a) the variation of NPP was mainly driven by flooding stress caused by DRW cycles; (b) NUE was one of the most critical predictors of NPP in the riparian zone ecosystem.

## 2 MATERIALS AND METHODS

### 2.1 Study area

The Pengxi River is one of the main tributaries of Yangtze River within TGR (30°49′30″‐ 31°41′30″N, 107°55′48″‐ 108°54′48″E). The riparian zone in the Pengxi River is one of the largest riparian zones of about 80 km^2^, accounting for 22.93% of the total riparian zone area, and its slope gradient is <15^o^ (Shi et al., 2017). The soil types are purple and yellow soil (Huang et al., 2017).

### 2.2 Sampling and analyzing

We investigated soil properties and vegetation distribution at 145–155 m, 155–165 m, and 165–175 m altitudes in the upper and downstream hydrological sections of Pengxi River (i.e., Qukou and Shuangjiang in Table 1) in July 2017 (Lin et al., 2018). Surface soils (0–20 cm) and corresponding aboveground vegetation were randomly collected. Each sample consisted of 3 random sampling plots (1 × 1 m) at each altitude. Hence, there were 18 soil samples and 18 plant samples in total (2 sites × 3 altitudes × 3 replicates). All samples were immediately stored in a 5°C incubator and then brought back to the laboratory within 24 hr. After removing plant residues, soil samples were passing through a 2‐mm sieve, air drying. The vegetation was preserved after washing and stoving at 75°C. Samples were ground to pass a 100 mesh before chemical analysis. The C and N were analyzed on an Elemental Analyzer (Euro Vector EA3000, Italy) equipped with Callidus software. The total phosphorus (P) was determined by the alkali fusion‐Mo‐Sb antispectrophotometric method. Soil ammonium (NH_4_^+^) and nitrate ( ${\text{NO}}_{3}^{-}$ ) contents were extracted by 1 M KCl solution and measured using the spectrophotometric method. Soil pH was measured by a standard pH meter after pretreatment with a 1:5 soil/water mass ratio. The soil texture was quantified using the hydrometer method (Gee & Bauder, 1979).

**Table 1:** The GPS coordinates of sampling points (Q: Qukou; S: Shuangjiang)

Transects	Name	Altitude (m)	Coordinates (°)
Qukou	Q1‐1	145–155	108.4940009230956,31.13550101049795
	Q1‐2	155–165	108.4938114238348,31.13532243094919
	Q1‐3	165–175	108.4936875151676,31.13514465485423
	Q2‐1	145–155	108.4972326105124,31.13434809505931
	Q2‐2	155–165	108.4970855260826,31.13408833535761
	Q2‐3	165–175	108.4969226349017,31.1337998431844
	Q3‐1	145–155	108.501406455594,31.13410619305287
	Q3‐2	155–165	108.5014587418277,31.1343233267925
	Q3‐3	165–175	108.5015371781648,31.13464401540984
Shuangjiang	S1‐1	145–155	108.69572799023,30.95805985727613
	S1‐2	155–165	108.69551243001,30.95798902485354
	S1‐3	165–175	108.6952597890038,30.95786795282588
	S2‐1	145–155	108.7018156241142,30.9559117748198
	S2‐2	155–165	108.7021645441206,30.95578479213048
	S2‐3	165–175	108.7025029937842,30.95565027453343
	S3‐1	145–155	108.6952590122355,30.94665507293436
	S3‐2	155–165	108.6956122129181,30.94658863833727
	S3‐3	165–175	108.6960016067077,30.94640569335657

### 2.3 Data evaluation

Net primary production (NPP) was determined from the changes in plant biomass (*W*) over a given time interval (Roberts, Long, Tieszen, & Beadle, 1985):

(1) $$P_{n}=\frac{\Delta W}{\Delta t}$$

**Figure 1:**
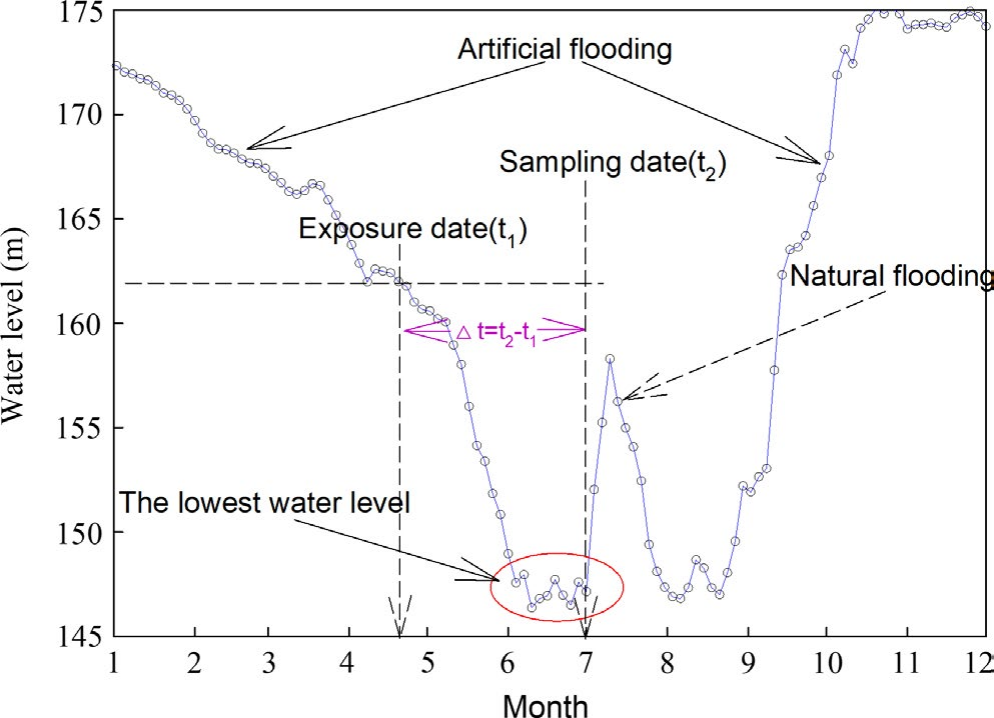
Monthly hydrologic records in 2017

*P_n_* is the net primary production of *C. dactylon* (g·m^−2^ day^−1^); ∆*W*_max_ is the change of biomass (g/m^2^); ∆*t* is the time interval from the exposure date (t_1_) of the specific riparian zone to sampling date (t_2_) for plant growth; t_1_ was obtained from the monthly hydrologic records in 2017 (Figure 1). It was assumed that plant begin to grow when the riparian zone exposure and no death occurs when the biomass is gained.

Nutrient (N, P) use efficiency (NUE) was a ratio of photosynthetic C gains per unit of N or P invested. Thus, the C: N or C: P ratio of individual biomass was regarded as a proxy measure of NUE in this study (Castellanos et al., 2018).

### 2.4 Data acquisition and statistical analysis

The flooding time was extracted from the water level data from 2014 to 2018 by GetData (V2.20) software with a 3 m water level interval. One‐way ANOVA was used to examine the differences in soil and plant properties at each altitude in the riparian zone. Linear regression was used to identify the relationship of NPP with soil and plant properties. All statistical analyses and plots were performed using SPSS 20.0 for Windows and SigmaPlot 12.5, respectively. Structure equation modeling (SEM) was conducted to judge the comprehensive effects (direct and indirect effect) of environmental factors and soil nutrimental properties on the response of plant NPP to flood stress. The chi‐square (χ^2^) test was used to assess the overall goodness of fit for the model. Nonsignificant χ^2^ test (*p* > .05), low values of χ^2^, RMSEA, and AIC, and χ^2^/*df* within 0–2 indicate the model is acceptable (Schermelleh‐Engel, Moosbrugger, & Müller, 2003) and suggest that there is a small difference between the observed and modeled values. All data were tested for normality using the Kolmogorov–Smirnov test, and non‐normal data were log‐transformed (e.g., soil C:N, C:P, and N:P). IBM SPSS Amos 24 was used to perform SEM.

## 3 RESULTS

### 3.1 Effect of environmental properties on NPP

**Figure 2:**
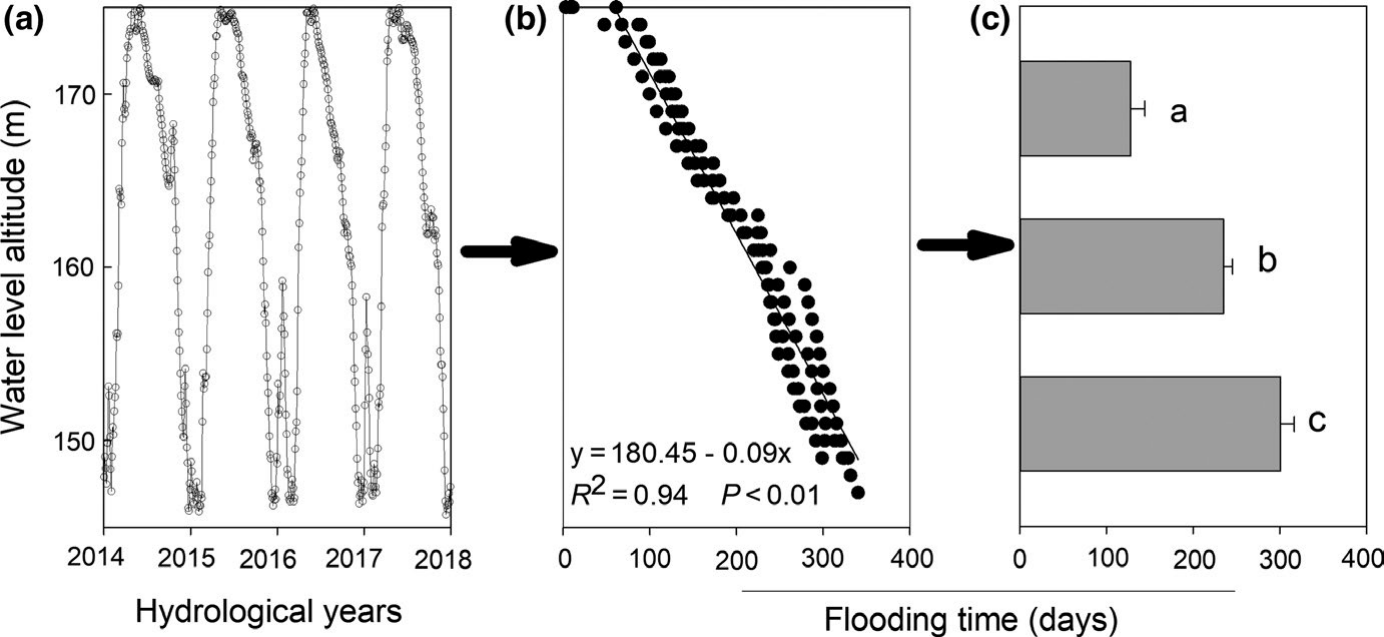
Water level fluctuation (a), relationships of altitudes with flooding time (b) flooding time of 145–155, 155–165, and 165–175 m altitudes (c)

**Figure 3:**
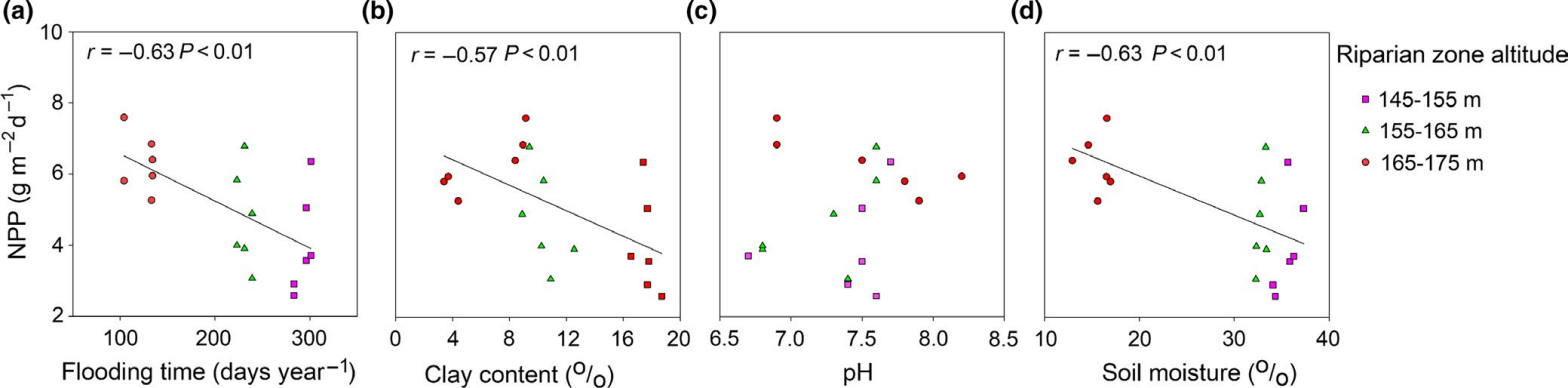
Relationships of NPP with environmental properties (flooding stress, pH, moisture, and clay content) in the riparian zone. Both correlation coefficients (*r*) and the associated *p* values were for this study

Seasonal changes in the water level were determined by hydrologic records from 2014 to 2018 from the Wanzhou Hydrological Station on the Yangtze River. Due to the hydrologic regime operation, the submerge time was different among the water level altitudes of the riparian zone of TGR, which reached about 300, 227, and 128 d at altitudes of 145–155, 155–165, and 165–175 m, respectively (Figure 2). NPP decreased with the increasing of flooding time (*r *= −.63, *p* < .01; Figure 3a), but it did not show any significant correlation with pH (Figure 3c). Both soil clay (*r* = −.57, *p* < .01; Figure 3b) and moisture content (*r* = −.63, *p* < .01; Figure 3d) were negatively correlated with NPP.

### 3.2 Effect of soil nutrients on NPP

**Figure 4:**
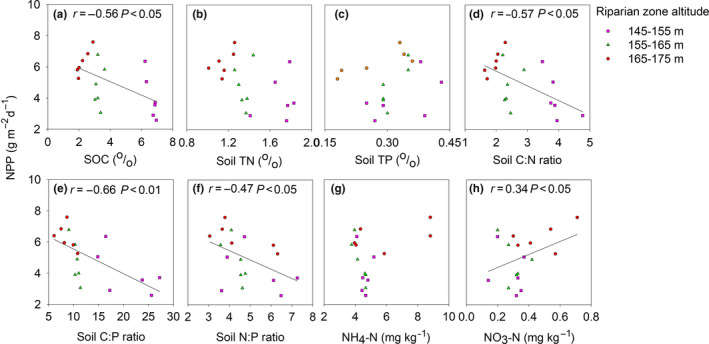
Relationship of the NPP with soil nutrients (SOC, TN, TP, NH_4_‐N, NO_3_‐N, C:N, C:P, N:P ratio). Both correlation coefficients (*r*) and the associated *p* values were for this study

Soil C, N, P, C:N, C:P, and N:P ratio decreased, while NH_4_^+^ and NO_3_^‐^ increased with the rising of riparian zone altitudes. Moreover, pH had no significant differences among altitudes (Table 2). NPP was negatively related to soil C (*r* = −.56, *p* < .05; Figure 4a), while positively related to NO_3_‐*N* (*r* = .34, *p* < .05; Figure 4h). In addition, NPP was negatively correlated with C:*N* (*r* = −.57, *p* < .05; Figure 4d), C:P (*r* = −.66, *p* < .01; Figure 4e), and N:P ratio (*r* = −.47, *p* < .05; Figure 4f). However, NPP was not significantly correlated with soil TN, TP, and NH_4_‐*N* (Figure 4b,c,g).

**Table 2:** Soil properties (*n* = 6, ±*SD*)

Items	Riparian zone altitudes		
	145–155 m	155–165 m	165–175 m
SOC (^o^/_o_)	6.67 ± 0.31a	3.25 ± 0.23a	2.25 ± 0.39b
TN (^o^/_o_)	1.71 ± 0.15a	1.35 ± 0.06a	1.15 ± 0.09b
TP (^o^/_o_)	0.33 ± 0.07a	0.31 ± 0.02a	0.27 ± 0.07b
C:N	3.94 ± 0.44a	2.41 ± 0.25a	1.94 ± 0.24b
C:P	20.86 ± 5.29a	10.46 ± 0.80a	8.52 ± 1.69b
N:P	5.35 ± 1.49a	4.50 ± 0.44b	4.35 ± 1.36b
pH_CaCl2_	7.40 ± 0.36a	7.31 ± 0.41a	7.46 ± 0.56a
${\text{NH}}_{4}^{+}$ (mg/kg)	4.30 ± 0.37a	4.61 ± 0.41ab	5.97 ± 2.31b
${\text{NO}}_{3}^{-}$ (mg/kg)	0.28 ± 0.09a	0.30 ± 0.07b	0.47 ± 0.16c
Clay (%)	17.64 ± 0.69a	10.40 ± 1.28b	6.72 ± 2.91c
Silt (%)	20.75 ± 1.44a	17.93 ± 3.50a	10.18 ± 2.32b
Sand (%)	61.54 ± 1.17a	71.67 ± 4.51b	82.90 ± 4.77c
Soil moisture (%)	35.59 ± 1.21a	32.82 ± 0.47a	15.53 ± 1.71b

Note

Different alphabets indicate significant differences (*p* < .05) among the altitudes

### 3.3 Relationship between NPP and NUE

**Figure 5:**
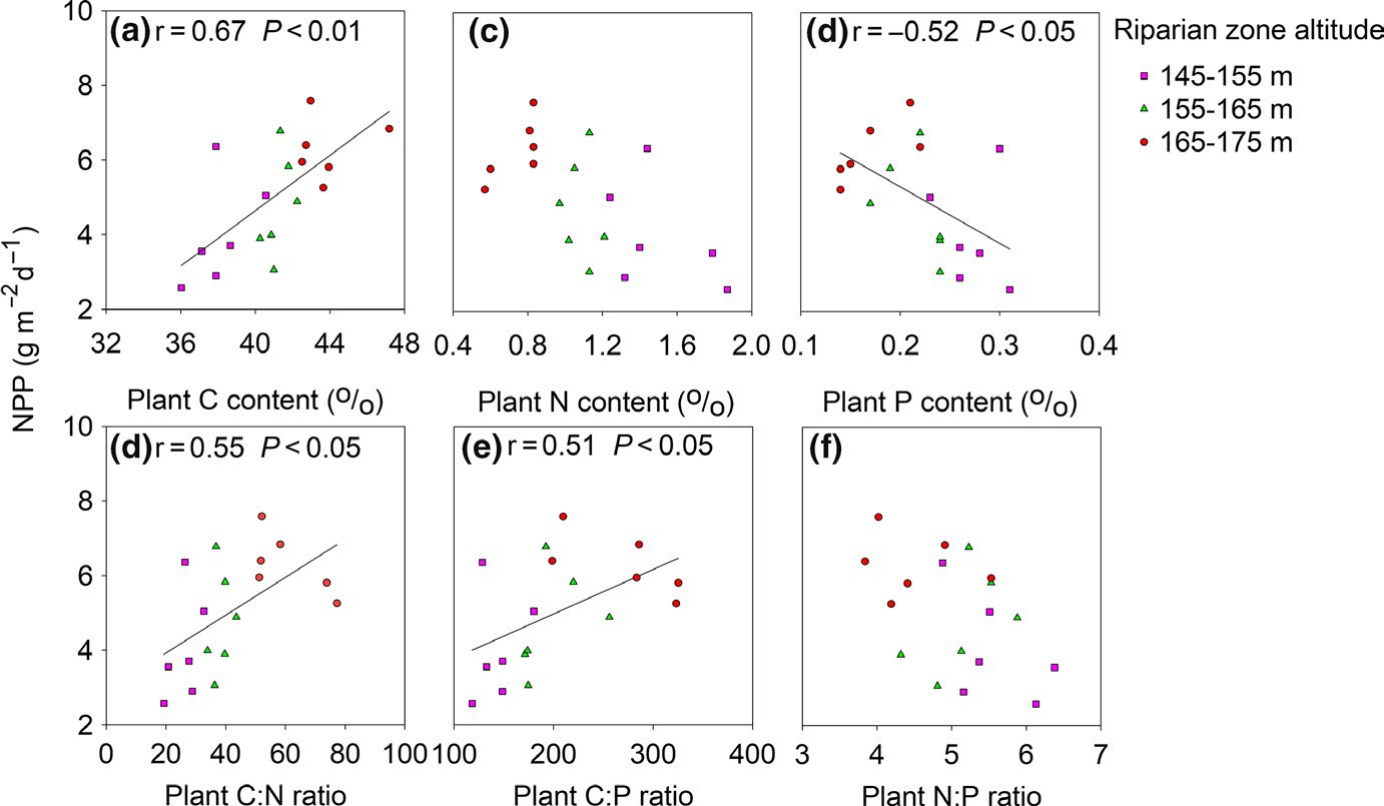
Relationship of NPP with nutrients in individual biomass. Both correlation coefficients (*r*) and the associated *p* values were for this study

The nutrients and biomass of *C. dactylon* significantly changed with the increase of flooding stress (Table 3). Compared with the higher altitudes, *C. dactylon* grew on the lower altitude of the riparian zone had lower C content, C:N and C:P, and on the contrary, much higher N and P content and N:P ratio. The biomass of *C. dactylon* decreased with the decline of altitudes, shown as 165–175 m > 155–165 m > 145–155 m. NPP was much higher when they contained much higher C, C:N and C:P ratio, and much lower P (Figure 5).

**Table 3:** Plant properties (*n* = 6, ±*SD*)

Items	Riparian zone altitudes		
	145–155 m	155−165 m	165−175 m
C (^o^/_o_)	38.03 ± 1.53a	41.24 ± 0.71ab	43.82 ± 1.73b
*N* (^o^/_o_)	1.51 ± 0.26a	1.08 ± 0.09a	0.75 ± 0.13b
P (^o^/_o_)	0.27 ± 0.03a	0.22 ± 0.03ab	0.17 ± 0.04b
C:N	25.94 ± 5.06 a	38.32 ± 3.39b	60.72 ± 11.80c
C:P	142.78 ± 21.87a	197.94 ± 33.85ab	271.00 ± 54.90b
N:P	5.57 ± 0.58 a	5.15 ± 0.55 ab	4.48 ± 0.63b
Biomass (kg/m^2^)	0.19 ± 0.07a	0.85 ± 0.59b	1.62 ± 0.81c

Note

Different alphabets in the same row mean significant differences at *p* < .05.

### 3.4 Exploring the drivers of NPP

**Figure 6:**
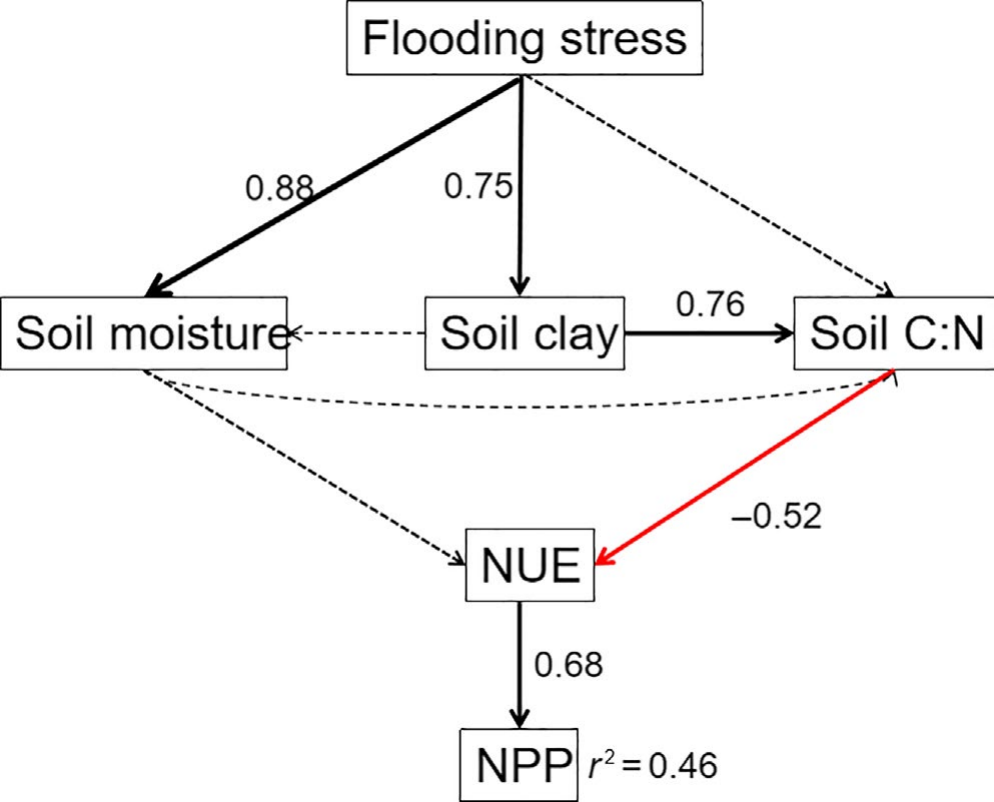
Structural equation modeling with variables (boxes) and potential causal relationships (arrows) for NPP. The red‐headed arrows indicate that the hypothesized direction of causation is a negative relationship; on the contrary, the black‐headed arrows represent a positive relationship. Arrow width is proportional to the strength of path coefficients. Gray dashed lines represent the relationships among variables that may exist but that not related significantly in this study. Standardized path coefficients (numbers) can reflect the importance of the variables within the model (Colman & Schimel, 2013). The model for NPP had χ^2^ = 13.7, *df* = 7, *p* = .053, RMSEA = 0.226 and AIC = 55.688. Clay, soil clay content; NPP, net primary production; NUE, nutrient use efficiency

**Figure 7:**
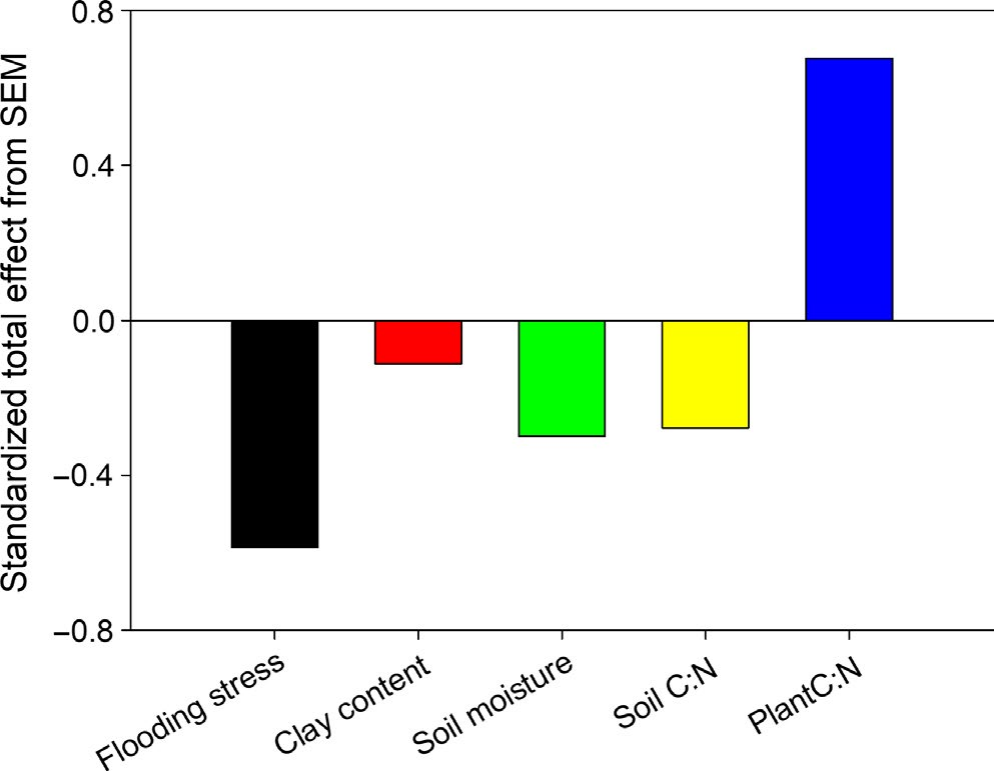
Standardized total effects (direct effect plus indirect effect) on NPP derived from the structural equation modeling (SEM). Clay, soil clay content; NPP, net primary production; NUE, nutrient use efficiency

Structure equation modeling analysis showed that flooding time, soil moisture, soil clay content, and soil C: N ratio had indirect effects, whereas NUE exerted direct effects. All of these variables predicted 46% of the variance in the NPP (Figure 6). Specifically, flooding stress had a direct positive effect on soil moisture and soil clay content. The clay content positively affects the soil C: N ratio, which further had an indirect negative effect on NPP by mediating NUE. Taking the total effect of direct and indirect effects into account, NUE was one of the most crucial predictors shaping the spatial pattern of NPP (Figure 7).

## 4 DISCUSSION

### 4.1 Effect of DRW cycles on environmental properties and NPP

The hydrological operation in the TGR, which had brought about antiseasonal DRW cycles since 2003, significantly influenced environmental properties in the riparian zone of Three Gorges tributaries. The environmental properties, such as flooding time, soil clay content, and soil moisture in the riparian zone, indirectly influenced on NPP of *C. dactylon*. Soil clay and moisture content were found much higher on the lower altitude (Table 2), which was directly related to the flooding time (Figure 6). NPP was negatively related to soil clay and soil moisture (Figure 3). The increasing of clay content at the low altitude of the riparian zone may come from higher annual average sedimentation rates, which was significantly decreased with increasing elevation (Tang et al., 2014). It is indicated that the mean sedimentation rates were 5.8 cm/year at the altitude of 145–150 m and 2.3 cm/year at the altitude of 150–175 m during 2007–2009 (Bao, Nan, He, Long, & Zhang, 2010). Compared with anthropogenic disturbance (2.1%), the variation in soil properties was mainly controlled by water level fluctuation (40.1%) (Ye et al., 2019). The flooding duration was significantly negatively correlated with the altitudes of the riparian zone (Figure 2). Periodical flooding stresses may enlarge the loss of biodiversity and reduce soil structure stability, and then lead to soil aggregates destruction and soil erosion increase (Ran et al., 2020; Wang et al., 2015). The maximum soil erosion rate happened in the conventional tillage farmland, and on the contrary, the minimum soil erosion rate was found in the natural and artificial grassland (Bao, He, Wei, Tang, & Guo, 2012). It is thus clear that soil erosion was varied with different land use and management practices. However, in this study, we just focused on the natural grassland, which was dominated by *C. dactylon* and impacted less by soil erosion, for exploring relative contribution of environmental and nutritional properties to NPP. So, the contribution in other land uses need to further study. Besides, other drive forces might include water wave, gravity, and surface runoff (Bao, Tang, He, Hu, & Zhang, 2013). The difference in flooding duration made environmental properties significantly distinct among the altitudes. The combined effects of environmental factors determined the NPP in the riparian zone of TGR. Thus, DRW cycles were one of dominating limiting factors regulating environmental properties and NPP in the riparian zone ecosystem.

### 4.2 Flooding stress on NPP

The SEM indicated that flooding stress was the main driver of NPP (Figure 6), which supported our first hypothesis. NPP was negatively related to flooding time (*p* < .01, Figure 3a) and soil moisture (*p* < .01, Figure 3d). During flooding, excessive moisture infiltration could restrict seed germination, and thus, most plants have difficulty surviving or recovering after submergence (Riis & Hawes, 2002; Sun & Wang, 2016). The respiration, photosynthesis, growth, and development of plants may also be restricted with the changes of environmental factors, such as illumination, oxygen content, and water pressure (Banach et al., 2009). The previous observations showed that NPP of *Populus euphratica* were lower than that nonflooded by reducing leaf gas exchange, and thus decrease total chlorophyll content, on the contrary, increasing the leaf soluble sugar in the riparian zone along the Tarim River (Yu, Zhao, Li, Li, & Peng, 2015). To adapt to flooding stress, *Phalaris arundinacea* took escape strategy with higher NPP, and on the contrary, *Carex cinerascens* make use of a more quiescence strategy, with less functional traits responses (Lan et al., 2019). Water‐use efficiency might be another essential factor for NPP. The reduced NUE caused by water deficits may be compensated by the increasing of water‐use efficiency associated with stomatal closure in the short‐term (Maroco, Pereira, & Manuela Chaves, 2000). Besides, continuous flooding could enhance biomass production of *Typha latifolia*. On the contrary, periodic drought led to the biomass reduction in floodplain ecosystems across North America (Li, Pezeshki, & Goodwin, 2004). The lower biomass of *C. dactylon* was found in the riparian zone of 145–155 m altitude with much longer flooding duration (Table 3). Thus, NPP and NUE might depend on physiological and genetic properties for different species, while that for one species that grew in a specific habitat may depend on the environmental properties and water‐use efficiency.

### 4.3 Effect of soil nutrition use efficiency on NPP

The change of environmental factors controlled the distribution of soil nutrients along the riparian zone that closely related to flooding duration. The nutrients including P, available P, available potassium, and nitrate decide the distribution and growth of plant species along the riparian zone (Ye, Zhang, Deng, & Zhang, 2013). Much higher soil moisture and clay content were associated with much higher SOC, TN, TP, C:N ratio, C:P ratio, N:P ratio, and much lower NH_4_^+^ and NO_3_^‐^ in the riparian zone (Table 2). NPP has a negative correlation with SOC, soil C:N ratio, soil C:P ratio, and soil N:P ratio, while a positive correlation with NO_3_‐*N* (Figure 4). Plant roots that mostly distributed in the surface layer (0–10 cm) have significant effects on the distribution of soil nutrients in soil profiles in the riparian zone (Zhong, Hu, Bao, Wang, & He, 2018). P pool was formed by continuous periodic flooding and associated with the deposition of fine particles (Wu et al., 2016). Thus, all of those factors could influence NUE. NUE was represented by C: N or C: P ratio of individual biomass usually varied with soil nutrient supply. Plants tend to maintain C: N: P stoichiometric balance as a survival strategy (Zhang, Su, & Yang, 2017). Meanwhile, perennials in the riparian zone had a significantly higher area‐based photosynthetic capacity with much higher stomatal conductance, leaf nitrogen concentration, and stem mass ratio than those in upland (Zhang, Fan, Li, Xiong, & Xie, 2016). Thus, it is essential for optimal growth by changing NUE strategies to survive under variable or unbalanced N and P availability. NPP was positively related to NUE (Figure 5d,e). The DRW cycles induced NUE changes could influence NPP by altering the patterns of growth, reproduction, and distribution. It has shown a plant‐mediated survival strategy by changing C:N:P stoichiometry to sustain a suitable NPP under a nutrient‐deficient condition (Castellanos et al., 2018). Therefore, NUE is a crucial predicting factor for NPP.

## 5 CONCLUSIONS

Based on SEM, we showed an important consequence of DRW cycles on NPP by changing NUE strategy. All of the environmental and nutritional variables could predict 46% of the NPP variance. NUE was one of the most critical predictors for shaping the change of NPP. Overall, this study provided a comprehensive analysis of the effects of the combined effect of environmental and nutrient factors on NPP and showed that continuous DRW cycles induced by hydrological regime stimulate the decrease of NPP of *C. dactylon* by changing NUE strategies. Further research is needed to explore the responses of NPP and NUE under different land use to DRW cycles and to investigate the DRW effects on the combined effect of environmental and nutrient factors by in situ experiments and long‐term studies.

The authors declare that they have no known competing financial interests or personal relationships that could have appeared to influence the work reported in this paper.

## CONFLICT OF INTEREST

None declared.

## AUTHOR CONTRIBUTION

**Junjie Lin:** Funding acquisition (lead); Investigation (lead); Visualization (lead); Writing‐original draft (lead); Writing‐review & editing (lead). **Shuang Zhou:** Data curation (equal); Investigation (equal). **Dan Liu:** Data curation (equal); Investigation (equal); Methodology (equal). **Shuai Zhang:** Data curation (equal); Investigation (equal); Methodology (equal). **Zhiguo Yu:** Conceptualization (equal); Formal analysis (equal); Funding acquisition (equal); Writing‐review & editing (equal). **Xiaoxia Yang:** Conceptualization (equal); Investigation (equal); Writing‐review & editing (equal).

## Data Availability

All data are contained within the manuscript and its appendices.

## References

[ece36409-bib-0001] Bai, Z. , & Weijie, L. (2018). The ecological footprint and carrying capacity in Northeast Asia In Himiyama Y. (Ed.), Exploring sustainable land use in Monsoon Asia, Springer Geography book series (pp. 267–275). Singapore: Springer 10.1007/978-981-10-5927-8_14

[ece36409-bib-0002] Banach, A. M. , Banach, K. , Peters, R. C. J. H. , Jansen, R. H. M. , Visser, E. J. W. , Stępniewska, Z. , … Lamers, L. P. M. (2009). Effects of long‐term flooding on biogeochemistry and vegetation development in floodplains; a mesocosm experiment to study interacting effects of land use and water quality. Biogeosciences, 6, 1325–1339.

[ece36409-bib-0003] Bao, Y. , He, X. , Wei, J. , Tang, Q. , & Guo, F. (2012). Soil erosion under different land uses in the riparian zone of the Three Gorges Reservoir, China. IAHS Publications, 356, 198–201.

[ece36409-bib-0004] Bao, Y. , Nan, H. , He, X. , Long, Y. , & Zhang, X. (2010). Sedimentation in the riparian zone of the Three Gorges Reservoir, China In Sediment dynamics for a changing future (pp. 224–228). Wallingford, UK: IAHS Publication. International Association of Hydrological Sciences.

[ece36409-bib-0005] Bao, Y. , Tang, Q. , He, X. , Hu, Y. , & Zhang, X. (2013). Soil erosion in the riparian zone of the Three Gorges Reservoir, China. Hydrology Research, 46, 212–221.

[ece36409-bib-0006] Castellanos, A. E. , Llano‐Sotelo, J. M. , Machado‐Encinas, L. I. , López‐Piña, J. E. , Romo‐Leon, J. R. , Sardans, J. , & Peñuelas, J. (2018). Foliar C, N, and P stoichiometry characterize successful plant ecological strategies in the Sonoran Desert. Plant Ecology, 219, 775–788.

[ece36409-bib-0007] Chadha, A. , Florentine, S. K. , Chauhan, B. S. , Long, B. , & Jayasundera, M. (2019). Influence of soil moisture regimes on growth, photosynthetic capacity, leaf biochemistry and reproductive capabilities of the invasive agronomic weed, *Lactuca serriola* . PLoS One, 14, e0218191 10.1371/journal.pone.0218191 31251746 PMC6599151

[ece36409-bib-0008] Chen, X. , Zhang, S. , Liu, D. , Yu, Z. , Zhou, S. , Li, R. , … Lin, J. (2019). Nutrient inputs from the leaf decay of *Cynodon dactylon* (L.) *Pers* in the water level fluctuation zone of a Three Gorges tributary. Science of the Total Environment, 688, 718–723. 31255809 10.1016/j.scitotenv.2019.06.357

[ece36409-bib-0009] Colman, B. P. , & Schimel, J. P. (2013). Drivers of microbial respiration and net N mineralization at the continental scale. Soil Biology and Biochemistry, 60, 65–76.

[ece36409-bib-0010] Gee, G. , & Bauder, J. (1979). Particle size analysis by hydrometer: A simplified method for routine textural analysis and a sensitivity test of measurement parameters. Soil Science Society of America Journal, 43, 1004–1007.

[ece36409-bib-0011] Gholz, H. L. (1982). Environmental limits on aboveground net primary production, leaf area, and biomass in vegetation zones of the Pacific Northwest. Ecology, 63, 469–481.

[ece36409-bib-0012] Guo, Y. , Yang, S. , Shen, Y. , Xiao, W. , & Cheng, R. (2018). Composition and niche of the existing herbaceous plants in the water‐level‐fluctuating zone of the Three Gorges Reservoir Area, China. Ying Yong Sheng Tai Xue Bao=. the Journal of Applied Ecology, 29, 3559–3568. 30460802 10.13287/j.1001-9332.201811.006

[ece36409-bib-0013] Huang, D. , Wang, D. , & Ren, Y. (2019). Using leaf nutrient stoichiometry as an indicator of flood tolerance and eutrophication in the riparian zone of the Lijiang River. Ecological Indicators, 98, 821–829.

[ece36409-bib-0014] Huang, Y. , Yasarer, L. M. W. , Li, Z. , Sturm, B. S. M. , Zhang, Z. , Guo, J. , & Shen, Y. (2017). Air–water CO2 and CH4 fluxes along a river–reservoir continuum: Case study in the Pengxi River, a tributary of the Yangtze River in the Three Gorges Reservoir, China. Environmental Monitoring and Assessment, 189, 223. 28429251 10.1007/s10661-017-5926-2

[ece36409-bib-0015] Lan, Z. , Huang, H. , Chen, Y. , Liu, J. , Chen, J. , Li, L. , … Chen, J. (2019). Testing mechanisms underlying responses of plant functional traits to flooding duration gradient in a lakeshore meadow. Journal of Freshwater Ecology, 34, 481–495.

[ece36409-bib-0016] Li, S. , Pezeshki, S. R. , & Goodwin, S. (2004). Effects of soil moisture regimes on photosynthesis and growth in cattail (*Typha latifolia*). Acta Oecologica, 25, 17–22.

[ece36409-bib-0017] Lin, J. , Zhang, S. , Liu, D. , Yu, Z. , Zhang, L. , Cui, J. , … Fu, C. (2018). Mobility and potential risk of sediment‐associated heavy metal fractions under continuous drought‐rewetting cycles. Science of the Total Environment, 625, 79–86. 29289009 10.1016/j.scitotenv.2017.12.167

[ece36409-bib-0018] Marcelis, L. , Heuvelink, E. , & Goudriaan, J. (1998). Modelling biomass production and yield of horticultural crops: A review. Scientia Horticulturae, 74, 83–111.

[ece36409-bib-0019] Maroco, J. P. , Pereira, J. S. , & Manuela Chaves, M. (2000). Growth, photosynthesis and water‐use efficiency of two C4Sahelian grasses subjected to water deficits. Journal of Arid Environments, 45, 119–137.

[ece36409-bib-0020] Piao, S. , Fang, J. , Zhou, L. , Zhu, B. , Tan, K. , & Tao, S. 2005 Changes in vegetation net primary productivity from 1982 to 1999 in China. Global Biogeochemical Cycles, 19(2), GB2027 10.1029/2004GB002274

[ece36409-bib-0021] Ran, Y. , Wu, S. , Zhu, K. , Li, W. , Liu, Z. , & Huang, P. (2020). Soil types differentiated their responses of aggregate stability to hydrological stresses at the riparian zones of the Three Gorges Reservoir. Journal of Soils and Sediments, 20, 951–962.

[ece36409-bib-0022] Riis, T. , & Hawes, I. (2002). Relationships between water level fluctuations and vegetation diversity in shallow water of New Zealand lakes. Aquatic Botany, 74, 133–148.

[ece36409-bib-0023] Roberts, M. J. , Long, S. P. , Tieszen, L. L. , & Beadle, C. L. 1985 CHAPTER 1 ‐ Measurement of plant biomass and net primary production In Coombs J., Hall D. O., Long S. P., & Scurlock J. M. O. (Eds.) Techniques in bioproductivity and photosynthesis, 2nd ed (pp. 1–19). Oxford, UK: Pergamon 10.1016/b978-0-08-031999-5.50011-x

[ece36409-bib-0024] Ruimy, A. , Dedieu, G. , & Saugier, B. (1996). TURC: A diagnostic model of continental gross primary productivity and net primary productivity. Global Biogeochemical Cycles, 10, 269–285.

[ece36409-bib-0025] Schermelleh‐Engel, K. , Moosbrugger, H. , & Müller, H. (2003). Evaluating the fit of structural equation models: Tests of significance and descriptive goodness‐of‐fit measures. Methods of Psychological Research Online, 8, 23–74.

[ece36409-bib-0026] Shi, Y. , Xu, G. , Wang, Y. , Engel, B. A. , Peng, H. , Zhang, W. , … Dai, M. (2017). Modelling hydrology and water quality processes in the Pengxi River basin of the Three Gorges Reservoir using the soil and water assessment tool. Agricultural Water Management, 182, 24–38.

[ece36409-bib-0027] Sun, J. , & Wang, H. (2016). Soil nitrogen and carbon determine the trade‐off of the above‐and below‐ground biomass across alpine grasslands, Tibetan Plateau. Ecological Indicators, 60, 1070–1076. 10.1016/j.ecolind.2015.08.038

[ece36409-bib-0028] Tang, Q. , Bao, Y. , He, X. , Zhou, H. , Cao, Z. , Gao, P. , … Zhang, X. (2014). Sedimentation and associated trace metal enrichment in the riparian zone of the Three Gorges Reservoir, China. Science of the Total Environment, 479, 258–266. 24561931 10.1016/j.scitotenv.2014.01.122

[ece36409-bib-0029] Wang, Q. , Yuan, X. , Willison, J. M. , Zhang, Y. , & Liu, H. (2014). Diversity and above‐ground biomass patterns of vascular flora induced by flooding in the drawdown area of China's Three Gorges Reservoir. PLoS One, 9, e100889 10.1371/journal.pone.0100889 24971514 PMC4074080

[ece36409-bib-0030] Wang, W. Q. , Sardans, J. , Wang, C. , Zeng, C. S. , Tong, C. , Asensio, D. , & Peñuelas, J. (2015). Ecological stoichiometry of C, N, and P of invasive *Phragmites australis* and native *Cyperus malaccensis* species in the Minjiang River tidal estuarine wetlands of China. Plant Ecology, 216, 809–822. 10.1007/s11258-015-0469-5

[ece36409-bib-0031] Wu, J. , Huang, J. , Han, X. , Gao, X. , He, F. , Jiang, M. , … Shen, Z. (2004). The three gorges dam: An ecological perspective. Frontiers in Ecology and the Environment, 2, 241–248.

[ece36409-bib-0032] Wu, Y. , Wang, X. , Zhou, J. , Bing, H. , Sun, H. , & Wang, J. (2016). The fate of phosphorus in sediments after the full operation of the Three Gorges Reservoir, China. Environmental Pollution, 214, 282–289. 27105164 10.1016/j.envpol.2016.04.029

[ece36409-bib-0033] Yan, Z. , Kim, N. , Han, W. , Guo, Y. , Han, T. , Du, E. , & Fang, J. (2015). Effects of nitrogen and phosphorus supply on growth rate, leaf stoichiometry, and nutrient resorption of *Arabidopsis thaliana* . Plant and Soil, 388, 147–155.

[ece36409-bib-0034] Yang, F. , Liu, W.‐W. , Wang, J. , Liao, L. , & Wang, Y. (2012). Riparian vegetation’s responses to the new hydrological regimes from the Three Gorges Project: Clues to revegetation in reservoir water‐level‐fluctuation zone. Acta Ecologica Sinica, 32, 89–98. 10.1016/j.chnaes.2012.02.004

[ece36409-bib-0035] Ye, C. , Butler, O. M. , Chen, C. , Liu, W. , Du, M. , & Zhang, Q. (2020). Shifts in characteristics of the plant‐soil system associated with flooding and revegetation in the riparian zone of Three Gorges Reservoir, China. Geoderma, 361, 114015 10.1016/j.geoderma.2019.114015

[ece36409-bib-0036] Ye, C. , Li, S. , Zhang, Y. , & Zhang, Q. (2011). Assessing soil heavy metal pollution in the water‐level‐fluctuation zone of the Three Gorges Reservoir, China. Journal of Hazardous Materials, 191, 366–372. 21571427 10.1016/j.jhazmat.2011.04.090

[ece36409-bib-0037] Ye, C. , Zhang, K. , Deng, Q. , & Zhang, Q. (2013). Plant communities in relation to flooding and soil characteristics in the water level fluctuation zone of the Three Gorges Reservoir, China. Environmental Science and Pollution Research, 20, 1794–1802. 22968672 10.1007/s11356-012-1148-x

[ece36409-bib-0038] Ye, F. , Ma, M. H. , Wu, S. J. , Jiang, Y. , Zhu, G. B. , Zhang, H. , & Wang, Y. (2019). Soil properties and distribution in the riparian zone: The effects of fluctuations in water and anthropogenic disturbances. European Journal of Soil Science, 70, 664–673.

[ece36409-bib-0039] Yu, B. , Zhao, C. Y. , Li, J. , Li, J. Y. , & Peng, G. (2015). Morphological, physiological, and biochemical responses of *Populus euphratica* to soil flooding. Photosynthetica, 53, 110–117.

[ece36409-bib-0040] Zhang, A. , Fan, D. , Li, Z. , Xiong, G. , & Xie, Z. (2016). Enhanced photosynthetic capacity by perennials in the riparian zone of the Three Gorges Reservoir Area, China. Ecological Engineering, 90, 6–11.

[ece36409-bib-0041] Zhang, K. , Su, Y. , & Yang, R. (2017). Biomass and nutrient allocation strategies in a desert ecosystem in the Hexi Corridor, northwest China. Journal of Plant Research, 1–10. 28401322 10.1007/s10265-017-0940-6

[ece36409-bib-0042] Zhang, M. , Lal, R. , Zhao, Y. , Jiang, W. , & Chen, Q. (2017). Spatial and temporal variability in the net primary production of grassland in China and its relation to climate factors. Plant Ecology, 218, 1117–1133.

[ece36409-bib-0043] Zhong, R.‐H. , Hu, J.‐M. , Bao, Y.‐H. , Wang, F. , & He, X.‐B. (2018). Soil nutrients in relation to vertical roots distribution in the riparian zone of Three Gorges Reservoir, China. Journal of Mountain Science, 15, 1498–1509.

